# Avian Group D Rotaviruses: Structure, Epidemiology, Diagnosis, and Perspectives on Future Research Challenges

**DOI:** 10.3390/pathogens6040053

**Published:** 2017-10-24

**Authors:** Pallavi Deol, Jobin Jose Kattoor, Shubhankar Sircar, Souvik Ghosh, Krisztián Bányai, Kuldeep Dhama, Yashpal Singh Malik

**Affiliations:** 1 Division of Biological Standardization, ICAR-Indian Veterinary Research Institute, Izatnagar, Bareilly 243 122, India; pallavi.deol@gmail.com (P.D.); jobinjkattoor@gmail.com (J.J.K.); shubhankar.sircar@gmail.com (S.S.); 2 Department of Biomedical Sciences, One Health Center for Zoonoses and Tropical Veterinary Medicine, Ross University School of Veterinary Medicine, P. O. Box 334, Basseterre, St. Kitts, West Indies; souvikrota@gmail.com; 3 Institute for Veterinary Medical Research, Centre for Agricultural Research, Hungarian Academy of Sciences, Hungáriakrt. 21, Budapest 1143, Hungary; bkrota@hotmail.com; 4 Division of Pathology, ICAR-Indian Veterinary Research Institute, Izatnagar, Bareilly 243 122, India; kdhama@rediffmail.com

**Keywords:** rotavirus D, poultry, runting and stunting syndrome, diagnosis, epidemiology, control, challenges

## Abstract

In 1981, a new virus (virus 132) was described for the first time with morphological and biochemical similarities to rotaviruses (RVs), but without antigenic similarity to any of the previously known rotavirus groups. Subsequently, it was re-designated as D/132, and formed a new serogroup among rotaviruses, the group D rotavirus (RVD). Since their identification, RVs are the leading cause of enteritis and diarrhea in humans and various animal species, and are also associated with abridged growth, particularly in avian species. Recently, RVD has been suggested to play a role in the pathogenesis of runting and stunting syndrome (RSS), alongside other viruses such as reovirus, astrovirus, coronavirus, and others, all of which cause colossal economic losses to the poultry industry. RVD has been reported from several countries worldwide, and to date, only one complete genome sequence for RVD is available. Neither an immunodiagnostic nor a vaccine is available for the detection and prevention of RVD infection. Despite our growing understanding about this particular group, questions remain regarding its exact prevalence and pathogenecity, and the disease-associated annual losses for the poultry industry. Here, we describe the current knowledge about the identification, epidemiology, diagnosis, and prevention of RVD in poultry.

## 1. Introduction

The poultry production is one of the fastest expanding and most dynamically evolving divisions of the livestock sector worldwide. Globally, average poultry meat consumption is expected to rise by 3.7 kg and account for nearly 40% of meat consumed in 2030. A growth rate of 1.6 percent per year is expected in the world’s egg production, which projects an increment from 70.4 million metric tons in 2015 to 89.9 million metric tons in 2030. Improved breeding methods, nutrition, and management practices have led to this incredible achievement. However, selective breeding policies, intensive rearing, and the inadequate vaccination of birds are making them more vulnerable to diseases. Among the prevailing diseases, avian rotaviruses (AvRVs) are one of the important causes of viral enteric disease of poultry reported worldwide, and remain a threat to the poultry industry.

The human rotaviruses (RVs) were first identified in 1973 [1,2]. Years before 1973, several animal viruses, such as epizootic diarrhea of infant mice (EDIM) virus in mice [3], simian agent 11 (SA11) in vervet monkey [4], and 70 nm virus particles in diarrheic stools from calves [5], were described. All of these were later found to be RVs based on their morphology and group antigen sharing with human RVs [6]. Rotaviruses have been recognized as one of the main etiological agents of diarrhea and enteritis in mammals, including avian species [7,8]. Among avian species, RVs have been detected in turkeys, chickens, ducks, pigeons, pheasants, and wild birds [9,10,11,12,13,14,15]. Avian rotaviruses (AvRVs) were first identified from the United States (USA) in 1977 in turkey poults suffering from enteritis using electron microscopy, and afterwards in the United Kingdom (UK) in 1978 [16]. Since then, AvRVs have been reported from various geographical regions including France, Egypt, Argentina, Brazil, Nigeria, Bangladesh, and India [17,18,19]. As of now, RVs have been divided into eight serogroups, namely RVA, RVB, RVC, up to RVH (A–H), based on the gene composition and antigenic properties of the group-specific VP6 protein [20]. Lately, RVI in dogs and cats and RVJ in bats are designated as putative RV species, and are presently subjected for ICTV approval [21,22,23]. Among these, RVA, RVD, RVF, and RVG have been detected as AvRVs, with the predominance of RVA and RVD, while RVF and RVG are sporadic [24,25]. AvRVs cause colossal losses to the poultry industry, as they result in decreased feed adsorption, ultimately leading to reduced weight gain [8]. Apart from the gastrointestinal tract, RVs have also been detected in the pancreas and spleen of broilers with runting and stunting syndrome (RSS) [26]. Runting and stunting syndrome, which is caused by many different agents, including reovirus, astrovirus, coronavirus etc., has a highly negative impact on poultry sector. This disease is characterized by watery droppings, cloacal pasting, and the presence of diarrhea. Chicks are often undersized, pale, wet, and may have distended abdomens [27].

In 1994, the RVD-induced morphogenesis of enteric lesions was studied in pheasant chicks, and revealed that 66% of RVD-inoculated birds experienced diarrhea and stunted growth four days post-infection [28]. In 2006, RVD was identified as one of the causes in the pathogenesis of runting and stunting syndrome (RSS) [24]. Due to RSS, mortality and culling may reach up to 60%, and feed conversion may decrease 20 times, leading to huge economic losses [24,27]. One classic way to diagnose the RVD infection is through the detection of virus nucleic acid or antigen in intestinal contents or feces. Therefore, for diagnosis, conventional methods such as electron microscopy, polyacrylamide agarose gel electrophoresis (PAGE), and RT–PCR assay targeting the VP6 gene, are used [29]. Virus isolation is another approach, but is useful only for AvRVA, and has proved to be extremely difficult for the other RV species [7]. Although RVD infection has been documented in poultry birds, limited prevalence studies are available to date. The first detection of RVD was somewhere in early 1980s, where it was called virus 132, and rotavirus-like viruses (RVLV), and later re-designated as RVD [17,30,31]. Since then, RVD has been reported from many places, including a recent report from Nigeria [32]. This review compiles the available literature on RVD, including research regarding its genomic structure, epidemiology, diagnosis, and prevention.

## 2. Virus Structure and Genome

Complete rotavirus (RV) particles measure about 70 nm in diameter. The infectious RVs consist of a triple layered structure (TLP) that resembles a wheel (*lat. rota*), from which the name rotavirus was derived [33]. The innermost layer is formed by viral protein 2 (VP2), which encloses the 11 segmented dsRNA viral genome as well as the viral RNA-dependent RNA polymerase (RdRp), VP1, and the capping enzyme, VP3. The middle capsid layer is made up of VP6, which is a highly conserved, group-specific viral protein. The outermost layer is formed by VP4 (which is denoted as the ‘P’-Protease sensitive protein) and VP7 (denoted as the ‘G’-glycoprotein), against which the neutralizing and protective antibodies are generated in vitro and in vivo*,* respectively [34]. VP4 can undergo proteolytic cleavage, yielding two proteins, VP5 * and VP8 *, with the cleavage enhancing the infectivity of RVs [34].

**Figure 1:**
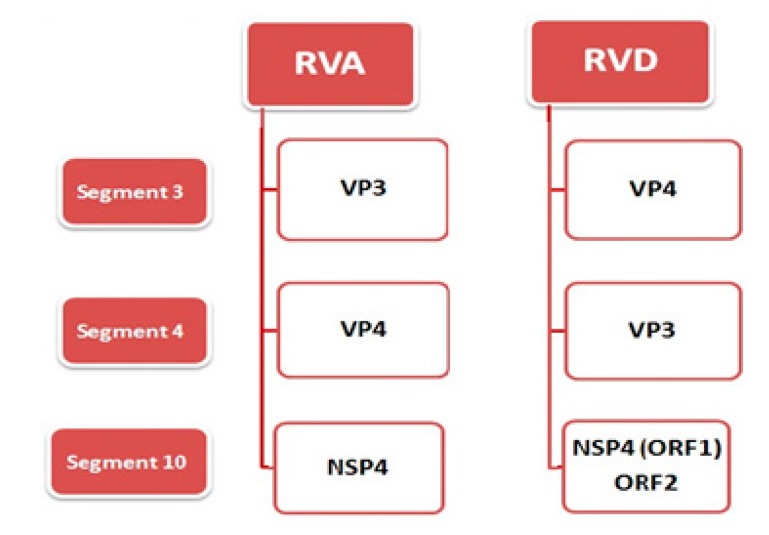
Notable differences among the gene products encoded by genome segments of two avian rotavirus groups, group D (RVD) and group A (RVA). The genome segments 3 and 4 encode for the VP3 and VP4 protein, respectively, in RVA, while the VP4 and VP3 proteins are encoded by genome segments 3 and 4 in RVD. Segment 10 carries one open reading frame (ORF) in RVA, while there are two in RVD.

The rotavirus genome consists of 11 segments of positive sense double-stranded (ds) RNA. The 11 segments of the RV genome are monocistronic, except for genome segment 11, which encodes two proteins in some serogroup of RVs. The molecular weight of this dsRNA ranges from 10^5^ to 10^6^ daltons, with a size range of 0.6–3.3 kilo base pairs, and an open reading frame (ORF) that encodes viral proteins is present in each RNA segment that encodes viral proteins [8,35,36]. The viral genome encodes six structural (VP1–VP4, VP6, VP7) and five/six non-structural proteins (NSP1–NSP5/6) [37,38]. Segment 1 encodes the VP1 protein and functions as a RNA-dependent RNA polymerase. It is located in the core of virion and complexed with VP3 protein, which in turn is encoded by segment 3. VP4, an outer capsid protein, is a major neutralizing antigen that is encoded by the fourth segment. Most of the serogroups of RVs follow the same rule, but variations have been found in some of the serogroups. In RVD, segment 3 encodes the VP4 protein, while segment 4 encodes the VP3 protein; this order is inverted compared with the gene–protein assignment of RVAs [39] (Table 1). Segment 10 of RVD has an additional open reading frame (ORF-2), which encodes a hypothetical protein [39]. The major differences between the genome organization of RVA and RVD are summarized in Figure 1.

Among the non-structural proteins (NSPs) of RVA, RVC, and RVD, the NSP2 protein shows the maximum sequence conservation. Avian RVA NSP1, which modulates the host immune response by mainly acting as an interferon antagonist, has shown more similarity to RVD than that of the mammalian RVA NSP1 sequence [39]. AvRVA NSP4, despite major differences in amino acid sequences analogized to NSP4 of mammalian RVs, acts as an enterotoxin, as did mammalian NSP4 proteins [37].

## 3. Classification

**Figure 2:**
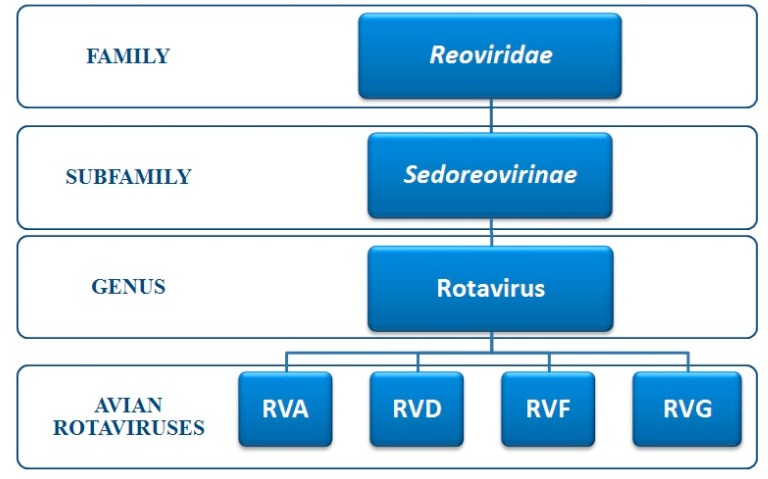
Classification of avian rotaviruses (AvRVs) within *Reoviridae* family*.*

Rotaviruses constitute the genus Rotavirus, which is one among 15 genera within *Reoviridae* family. *Reoviridae* is further subdivided into two sub-families, *Sedoreovirinae* and *Spinareovirinae* [6] (Figure 2).

Initially, cross-immunofluorescence studies or the polyacrylamide agarose gel electrophoresis (PAGE) analysis of dsRNA segments was used to classify RVs [36]. The antigenicity of RV is determined by three major structural proteins: VP4, VP6, and VP7. VP6 determines serogroups, whereas VP4 and VP7 determine serotypes. On the basis of the electrophoretic migration pattern of the RV genome segments and the antigenicity of inner capsid VP6 protein, eight different groups are defined by ICTV (designated as RVA–RVH) [20]. The putative serogroup RVI has been reported in sheltered dogs from Hungary and in cats from North America [21,22], while RVJ has been reported from bats in Serbia [23]. In 2008, for RVA, a nucleotide sequence-based classification system was adopted [40]. This system of classification assigns a specific genotype to each of the 11 RV genome segments according to established nucleotide percent cutoff values. The *VP7-VP4-VP6-VP1-VP2-VP3-NSP1-NSP2-NSP3-NSP4-NSP5/6* genes of RV strains are described using the abbreviations Gx-P[x]-Ix-Rx-Cx-Mx-Ax-Nx-Tx-Ex-Hx (x = Arabic numbers starting from 1), respectively [6]. As per the 10 June 2017 update of RCWG (Rotavirus Classification Working Group), the maximum number of P and G genotypes, P[50] and G35 respectively, have been reported. In India, the G6P[1], G6P[11], G6P[14], G3P[3], G8P[11], G10P[1], G10P[3], G10P[11] G10P[14], and G15P[21] genotypes of bovine RVA have been identified from different geographical locations [41]. There also have been attempts at creating genotype-based comprehensive classification systems for RVB and RVC [42,43,44,45]. RVs belonging to species A, B, and C (RVA, RVB, and RVC, respectively) infect humans and various animal species. RVs belonging to species D, E, F, and G (RVD, RVE, RVF, and RVG, respectively) have been recovered from animals only, so far.

**Figure 3:**
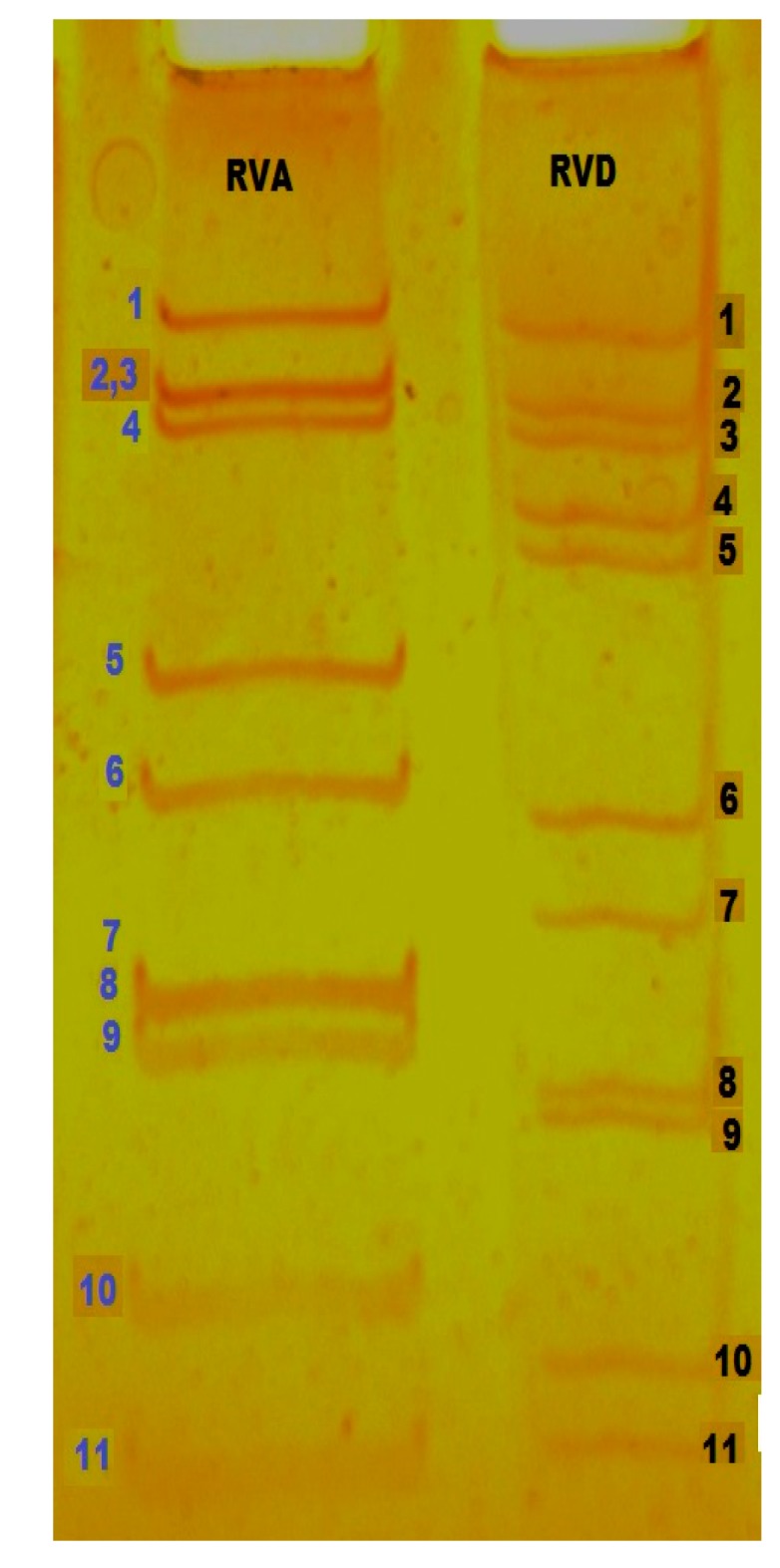
Electrophoretic migration pattern of mammalian type avian RVA and RVD in polyacrylamide gel (PAGE). Lane RVA: Mammalian type avian RVA with 4:2:3:2 and Lane RVD: RVD exhibit 5:2:2:2 genomic migration patterns.

Rotaviruses infecting avian spp. are designated as AvRVs. The RVs cross-reacting with antisera prepared against mammalian RVAs are classified as RVA, and those that lack the RVA antigen are referred to as atypical RVs/non-group A RVs that belong to groups D, F, and G, (designated as RVD, RVF, and RVG) which are exclusively found in poultry [29,31,32,36]. Although RVA is predominant across all host species, RVD is more common in poultry [46]. RVF and RVG have only been occasionally reported [47]. After analysis by PAGE, the genomic RNA segments initially clustered into four regions, I to IV. According to the distribution of segments in each region, the AvRV-A has a pattern of 5:1:3:2, and RV-D has a pattern of 5:2:2:2 (Figure 3), while mammalian type avian RV-A shows a pattern of 4:2:3:2 [17,30]. Reconstruction of the evolutionary history placed RVs in two major clades consisting of rotavirus A/C/D/F and rotavirus B/G/H [48]. This analysis is based on all six structural proteins and two (NSP2 and NSP5) of the five non-structural proteins. Within clade one, RVD is shown to be most closely related to RVF [48]. Studies based on codon usage of AvRVs done in 2015 suggested that there is a geographical preference in the usage of specific codons, i.e., the RVD nucleotide codons, for a particular protein, and that they are showing a selection process to adapt themselves to a particular geographical region [49]. Their study also revealed that the codon usage of AvRVs is more similar to that of yeast than human and *E. coli*, indicating that it may be easy to express RVD VP6 proteins through a yeast expression system.

## 4. Diversity of RVD across the World

The studies on RVD are flourishing, hence, novel sequence data are progressively updating in the public database. To date, only one whole genome data has been published [39] wherein the maximum number of annotations are available for the group specific protein gene, VP6. Comprehensive detail of all of the sequences available for the different genes is shown in Table 1 and Supplementary Table S1.

**Figure 4:**
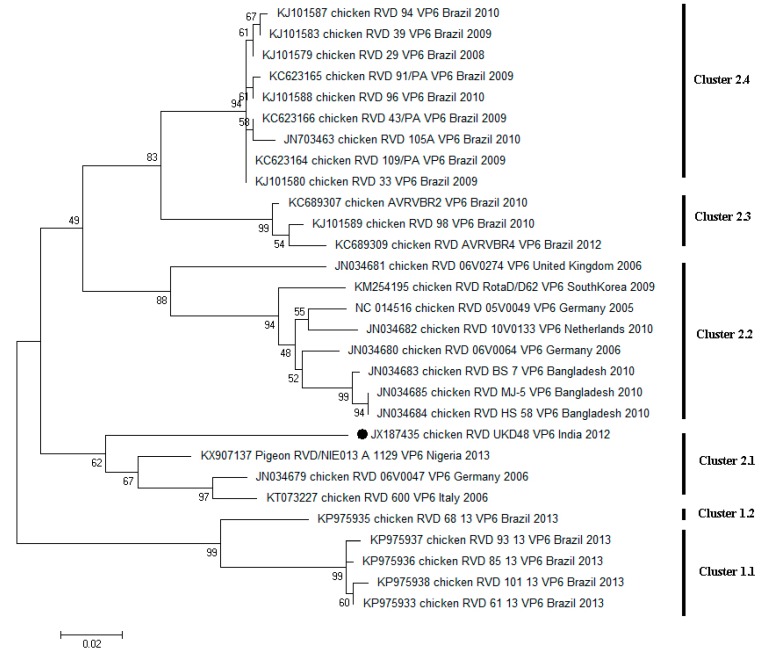
Dendrogram of the RVD VP6 gene constructed with maximum likelihood method based on the Tamura 3-parameter model. A discrete Gamma distribution was used to model evolutionary rate differences among sites. The strain (UKD48) analyzed in the study is shown with a black circle mark. Bootstrap values below 50 have been omitted. The tree is drawn to scale length representing 0.02 nucleotide substitutions per site.

The scarcity of gene sequences for study restricts RVD classification into different genotypes, as in the case of other group RVs. Nowadays, VP6 gene sequences have been used extensively by different researchers for the phylogenetic reconstruction of RVD isolates. Based on the VP6 gene sequence dendrogram topology, the geographical segregation of RVD isolates can be perceived. In the study done by Kattoor et al. (2013), a continent-specific clustering of sequences could be identified, where isolates from South America and Eurasia were showing a high content of divergence within the dendrogram [50]. In 2015, a study published by Beserra and coworkers revealed that the Brazilian isolates were forming exclusive clusters, and were well segregated from the rest of the world’s isolates [19]. However, as per our analysis, Brazilian sequences, which were collected at different time points, showed high divergence, which lead to the clustering of sequences into diverse clusters [Cluster 1.1, 2.3 and 2.4 (Figure 4)]. This observation is similar to that of European strains. In contrast, strains from Bangladesh were the least divergent among themselves (Figure 4).

## 5. Identification and Epidemiology

The first ever report of RVs in poultry was from the USA (South Dakota) in 1977 from intestinal contents of turkey poults [9]. In 1981, McNulty and coworkers isolated a virus (virus 132) from chickens of North Ireland. Despite having morphological and biochemical similarities to RVs, virus 132 was not antigenically related to any of the previously described RVs [30]. This virus was categorized as atypical RV/non-group A RV. Based on comparative antigenic and nucleic acid analysis, Pedley et al. designated this atypical RV/non-group A RV as D/132, and proposed the extension of the number of RV groups by including RVD as new member [31]. Later, Theil et al. (1986) found non-group A viruses from turkeys, which they called turkey rotavirus-like viruses (RVLVs) [17]. Likewise, RVLVs were also found in pheasants, and were later determined to be RVD [12]. Since then, RVD has been reported frequently in turkeys, chickens, and pheasants, and sporadically reported in guinea fowls, partridges, quails, pigeons, ducks, etc., [12,15,29]. Up to now, the prevalence and clinical importance of RVD has been recognized for chickens and turkeys in various countries. European countries from where RVD infections have been reported include Scotland, Sweden, Germany, the UK, Italy, and the Netherlands [29]. Apart from Europe, RVD has also been reported in chickens from the delta region of Egypt [18]. In 2012, a report on RVD infection from Brazil noted an incidence rate of 53% [51]. In the Asian continent, the first report on RVD was from Bangladesh [52]. After two years, a second report came from India, with an incidence of 17.39% [46]. A study conducted by Otto et al. (2012) [29] revealed a combined prevalence of 65.9% from Europe and Bangladesh [28]. Recently, in June 2017, nearly 32% of RVD shedding was observed in Nigerian birds. This study also suggested that host-permissive RVs may show cross-species transmission [32].

Although RVs cause enteric diseases in mammals and birds, they are often detected in otherwise healthy flocks, particularly when sensitive molecular diagnostic assays are used. Bezerra et al. (2014) reported the occurrence of RVD in apparently healthy asymptomatic chickens [51]. Mixed enteric infections have also been reported. Saif et al. (1990) isolated RVD, AsTV (astrovirus), Salmonella, and small round viruses (18–24 nm in diameter) from an enteritis outbreak in turkey poults [53]. RSS is a disease complex caused by many viruses, including reovirus, astrovirus, rotavirus etc. Four groups of AvRVs were recognized in flocks with RSS: avian RVA, RVD, RVF, and RVG. Otto et al. (2006) reported that RVD plays a major role in the pathogenesis of RSS in flocks with severe villous atrophy [24]. A recently granted patent also confirms that among all of the AvRVs, RVD plays a major role in the pathogenesis of RSS [54]. Earlier studies on rats have shown the capacity of AvRVs to disseminate and replicate in different organs, such as the liver, spleen, and pancreas; however, the mechanism by which RV escapes the gastrointestinal tract and reaches other organs remains unknown [55]. Recently, the extra-intestinal presence of avian RVA in the pancreas and spleen of broilers with RSS has also been reported [26]. However, there is no such report for RVD.

In India, the reports on RVD are very scarce. The first report on RVD came from central India in 2008, based on the specific electrophoretic migration pattern of RVD i.e., 5:2:2:2 [46]. In 2010, RVD was detected in western parts (Maharashtra) of India [56]. The first sequence of confirmed RVD was reported in broiler chicks from northern India in 2013 [50]. Subsequently, in 2014, an RT-PCR was developed for detection of RVD, with a better detection limit (1.49 × 10^3^ copies) than reported previously from Brazil [57]. Using this reverse transcription-polymerase chain reaction (RT-PCR), a frequency distribution study was conducted in 2011–2012, which confirmed the existence of RVD in chickens from northern states of India with a 6.04% positivity rate [58]. Using the same RT-PCR, recent (2012–2017) screening data revealed that this percent positivity in northern India has increased nearly fourfold over the last five years (Deol, unpublished data).

## 6. Diagnosis

Clinical manifestations of AvRV infection include mild to severe diarrhea, a varied degree of dehydration, and stunted growth; it also may remain asymptomatic. These variations may be due to the differences in the severity of the particular strain or the interaction between different environmental and management factors [36]. These manifestations alone are not sufficient for confirmatory diagnosis. Therefore, the identification of virus in fecal content or antibody detection in serum should be used to confirm the RV infection. To date, there is no assay for RVD antibody detection. The major methods used for diagnosis of RVs in poultry include:

*Electron microscopy*: The distinct wheel-like morphology of RV was initially used for detecting RV infection by direct visualization of the virus in feces or intestinal content [6]. Using this technique, RVs of different groups cannot be distinguished. Immune electron microscopy can be used to distinguish serogroups, although it requires the availability of specific antisera [59]. It is a sensitive diagnostic approach, but is also costly and cumbersome.

*Virus isolation:* AvRV can be isolated in embryonated chicken eggs (via yolk sac route), primary in cell culture (chicken embryo liver cells/chicken embryo kidney cells) or in continuous cell lines (MA104/Rhesus monkey kidney cell line) [17,60]. The isolation is useful only for AvRVAs, but it is not commonly used for diagnosis. It is very difficult to propagate other RV serogroups in cell cultures [6], and it has been reported that RVD cannot be propagated in MA104 cell culture systems [61].

*RNA Polyacrylamide Gel Electrophoresis (RNA-PAGE):* The detection of RV-RNA in feces or intestinal content provides an alternate means of diagnosis. Following RNA extraction, electrophoresis on polyacrylamide gels, and silver staining, RNA can be identified by the pattern of migration of genome segments. RNA-PAGE is a highly specific technique used for the detection of segmented viruses [35]. It detects the electrophoretic migration pattern of all 11 segments of RV, which is different among different groups. According to the distribution of segments in each region, the AvRV-A has a pattern of 5:1:3:2, and the RV-D has a pattern of 5:2:2:2, while the mammalian RV-A shows a pattern of 4:2:3:2. AvRVs RVF and RVG, which shows sporadic shedding, have migration patterns of 4:1:2:2:2 and 4:2:2:2:1, respectively [28]. However, these patterns can’t be totally relied upon, because substantial differences are observed in the electrophoretic pattern of RVs when conditions of gel electrophoresis are varied [62].

*Reverse Transcription-Polymerase Chain Reaction (RT-PCR):* This is the most sensitive molecular detection tool available for the diagnosis of RVs, and is mainly based on the VP6 gene segment [24,39,57]. For the detection of AvRVs, only a few RT-PCR protocols are available, and most of them solely detect AvRV-A [15]. In 2011, an RT-PCR was developed by Bezerra and co-workers specifically for the detection of the VP6 gene of RVD [63]. Real-time PCR and RT-PCR are now available for the detection of RVD. The sensitivity of real-time RT-PCR was found to be similar to that of conventional RT-PCR when the same primer sets were used for both of the assays [29].

*Serological methods:* Serological methods used for the detection of RVs include counter immunoelectrophoresis [64], radioimmunoassay (RIA) [65], the latex agglutination test (LAT) [66], and enzyme-linked immunosorbent assay (ELISA) [67]. AvRVs can be detected using ELISA. Commercialized ELISA kits are available for detection of RVAs, such as the IDEIA RV assay (DAKO, Ely, UK), the RIDASCREEN^®^ assay (r-biopharm, Darmstadt, Germany), etc. However, for the detection of other AvRVs such as RVD, RVF, or RVG, no ELISA is available.

## 7. Differential Diagnosis

Enteric infections are one of the principal causes of diarrhea, which results in poor feed conversion efficiency and reduced growth, and hence leads to heavy economic losses for the poultry industry. These infections are caused by different agents, including viruses (rotavirus, coronavirus, astrovirus, reovirus, adenovirus, parvovirus etc.), bacteria (Salmonella, Enterococcus, *E. coli)* and protozoans (Cryptosporidium and Eimeria) [68,69,70,71]. The clinical manifestations of these pathogens are almost similar. Hence, RVD must be differentiated from all of these enteric pathogens, as well as from the other groups of rotaviruses found in poultry.

## 8. Prevention

To date, it seems difficult to eliminate RVs from commercial flocks, as rotaviruses are ubiquitous as well as quite resistant to environmental conditions. The titre of AvRVs was found to decrease after treatment at 56 °C for 30 min, but the viral infectivity is difficult to inactivate completely [11,72]. Further, it was recognized that AvRVs were resistant to chloroform and were stable at pH 3. In general, sodium hypochlorite is an effective virucidal disinfectant, but for highly resistant AvRVs, glutaraldehyde had stronger activity, which is also true for enveloped viruses [73]. The spread of AvRVs can be prevented by maintaining good sanitation and hygiene by means of thorough cleaning and disinfection to reduce the environmental contamination between flocks [36]. Although RV disease is vaccine preventable, effective and safe vaccines are available only for human RVAs. As it is difficult to grow atypical AvRVs in cell culture, and also due to high antigenic variations among them, no vaccines are available so far for the prevention of AvRVs.

## 9. Conclusions and Future Prospects

The enteric viral infections, mainly rotavirus, are a global cause for concern. Among the four serogroups of rotaviruses identified in poultry, the RVD has gained more importance due to its involvement in runting and stunting syndrome. With the advent of molecular techniques, the diversity of RVD strains is beginning to be explored. However, still, the exact prevalence and annual losses associated with RVD in poultry industry are unknown. There are many gaps that need to be filled and warrant attention, such as:

▪ Studies on host–pathogen interactions, whether they are alike other enteric viruses or not.
▪ As RVD is found in both symptomatic and asymptomatic birds, factors responsible for its virulence and pathogenicity are to be studied.
▪ To date, very few sequences are available, and only for some of the genes of RVD strains. Once enough sequence data are available, a nucleotide sequence-based classification system can be established for RVD, as was achieved for RVAs.
▪ Only one complete genome sequence is available so far, despite the widespread distribution of RVD in chickens.
▪ The function of additional ORF (ORF-2) encoded by the 10th segment of RVD is still not defined.
▪ The development of sensitive and specific diagnostic tests, including the improvement of available ones, is of prime importance.
▪ The development of specific treatment by means of antivirals.

Once the basic information is available about RVD, its prevention should acquire the attention of researchers by means of developing vaccines to prevent its spread and to save one of the fastest-growing sectors, the poultry industry.

## Figures and Tables

**Table 1:** Comprehensive list of nucleotide sequences of RVD genes available in GenBank.

Gene Segment	RNA Segment Number Coding for the Gene	Size of Coding Sequence (in bp ^#^)	No. of Complete Sequences * (Accession No.)	No. of Partial Sequences	Total Number of Nucleotide Sequences
VP1	1	3237	1 (NC_014511)	4	5
VP2	2	2739	1 (NC_014512)	5	6
VP3	4	2055	2 (NC_014514, KF142491)	6	8
VP4	3	2331	1 (NC_014513)	6	7
VP6	6	1194	3 (NC_014516, KX374470, JX187435)	36	39
VP7	9	948	3 (KM254196, NC_014519, KF142489)	21	24
NSP1	5	1722	1 (NC_014515)	5	6
NSP2	8	930	1 (NC_014518)	3	4
NSP3	7	1110	1(NC_014517)	3	4
NSP4	10	ORF1: 381 ORF2: 279	4 (NC_014520,KF142490, KX374472, KX374471 )	3	7
NSP5	11	585	1 (NC_014521)	1	2

* Complete sequences include sequences with a complete specific protein-coding region. ^#^ base pair.

## Supplementary Materials

The following are available online at www.mdpi.com/2076-0817/6/4/53/s1, Table S1: The gene-wise (partial/complete) accession number details of avian rotavirus D.
