# Supplementary material for: Avian Group D Rotaviruses: Structure, Epidemiology, Diagnosis, and Perspectives on Future Research Challenges
Source: Pathogens. 2017 Oct 24;6(4):53. doi: 10.3390/pathogens6040053 (PMC5750577; doi:10.3390/pathogens6040053)
Supplement: Supplementary file 1 [file pathogens-06-00053-s001.pdf]

Supplementary Table S1. The gene-wise (partial/complete) accession number details of avian rotavirus D.

VP1 gene

Accession no	Complete/ Partial
NC014511	Complete
KM254191	Partial
KM254202	Partial
KY069074	Partial
KY069075	Partial

VP2 gene

Accession no	Complete/ Partial
NC014512	Complete
KM254203	Partial
KM254192	Partial
KY069078	Partial
KY069077	Partial
KY069076	Partial

VP3 gene

Accession no	Complete/ Partial
NC014514	Complete
KF142491	Complete
KM254204	Partial
KM254193	Partial

KY069081	Partial
KY069080	Partial
KY069079	Partial
KY069082	Partial

VP4 gene

Accession no	Complete/ Partial
NC_014513	Complete
KM254205	Partial
KM254194	Partial
KY069086	Partial
KY069084	Partial
KY069085	Partial
KY069083	Partial

VP6 gene

Accession no	Complete/ Partial
NC_014516	Complete
,KX374470	Complete
JX187435	Complete
JQ065736	Partial
JQ065735	Partial
JQ065734	Partial
KC623166	Partial
KC623165	Partial
KC623164	Partial
KX907137	Partial

KM254206	Partial
KM254195	Partial
KJ101589	Partial
KJ101588	Partial
KJ101587	Partial
KJ101586	Partial
KJ101585	Partial
KJ101584	Partial
KJ101583	Partial
KJ101582	Partial
KJ101581	Partial
KJ101580	Partial
KJ101579	Partial
KY069087	Partial
JX187434	Partial
JN703463	Partial
KC689309	Partial
KC689308	Partial
KC689307	Partial
KC689306	Partial
HM060261	Partial
HM060260	Partial
KP975938	Partial
KP975937	Partial
KP975936	Partial

KP975935	Partial
KP975934	Partial
KP975933	Partial
KT073227	Partial

VP7 gene

Accession no	Complete/ Partial
NC_014519	Complete
KF142489	Complete
KM254196	Complete
KM254207	Partial
KJ101578	Partial
KJ101577	Partial
KJ101576	Partial
KJ101575	Partial
KJ101574	Partial
KJ101573	Partial
KJ101572	Partial
KJ101571	Partial
KJ101570	Partial
KJ101569	Partial
KJ101568	Partial
KY069089	Partial
KY069088	Partial
KC669414	Partial
KC669413	Partial

KC669412	Partial
KC669411	Partial
KC669410	Partial
KC669409	Partial
KC669408	Partial

NSP1

Accession no	Complete/ Partial
NC_014515	Complete
KM254208	Partial
KM254197	Partial
KY069070	Partial
KY069069	Partial
KY069068	Partial

NSP2

Accession no	Complete/ Partial
NC_014518	Complete
KM254209	Partial
KM254198	Partial
KY069071	Partial

NSP3

Accession no	Complete/ Partial
NC_014517	Complete
KM254210	Partial

KM254199	Partial
KY069072	Partial

NSP4

Accession no	Complete/ Partial
NC_014520	Complete
KF142490	Complete
KX374472	Complete
KX374471	Complete
KM254211	Partial
KM254200	Partial
KY069073	Partial

NSP5

Accession no	Complete/ Partial
NC_014521	Complete
KM254201	Partial
